# Conformational dynamics of α-synuclein and study of its intramolecular forces in the presence of selected compounds

**DOI:** 10.1038/s41598-023-46181-1

**Published:** 2023-11-03

**Authors:** Zahed Khatooni, Keivan Akhtari, Heather L. Wilson

**Affiliations:** 1 Vaccine and Infectious Disease Organization (VIDO), University of Saskatchewan, Saskatoon, SK S7N 5E3 Canada; 2 Department of Physics, University of Kurdistan, P.O. Box 416, Sanandaj, Iran; 3 Department of Veterinary Microbiology, Western College of Veterinary Medicine, University of Saskatchewan, Saskatoon, SK S7N 5B4 Canada; 4 School of Public Health, Vaccinology & Immunotherapeutics Program, University of Saskatchewan, Saskatoon, SK S7N 5B4 Canada

**Keywords:** Biophysics, Computational biology and bioinformatics, Drug discovery, Genetics, Structural biology

## Abstract

Protein misfolding and aggregation play crucial roles in amyloidogenic diseases through the self-assembly of intrinsically disordered proteins (IDPs) in type II diabetes (T2D), Alzheimer's disease (AD) and Parkinson’s disease (PD). PD is the most common neurodegenerative disorder after AD, and is associated with the loss of dopaminergic signaling, which causes motor and nonmotor signs and symptoms. Lewy bodies and Lewy neurites are common pathological hallmarks of PD that are mainly composed of aggregates of disordered α-synuclein (α-Syn). There have been many efforts to develop chemical compounds to prevent aggregation or facilitate disruption of the aggregates. Furthermore, the roles and interactions of many compounds have yet to be revealed at the atomistic level, especially their impacts on the dynamics and chain-chain interactions of the oligomers, which are of interest in this study. The conformational diversity and detailed interactions among homo-oligomer chains of α-Syn are not fully discovered; identifying these might help uncover a practical approach to developing a potent therapy. In this study, we used an in-silico investigation to address the conformational diversity of α-Syn oligomer. The roles of several point mutations in protein aggregation in PD are known; we take this further by evaluating the interaction energies and contributions of all residues in stability and residue-chain interactions. In this study, we docked chemical derivatives of three compounds with high drug-likeness properties to evaluate the roles of our ligands in the conformational dynamicity of the oligomers, with emphasis on intramolecular forces. Free energy evaluation of the modeled inter and intramolecular interactions through MD simulation shows effective interaction and binding between α-Syn and our compounds. However, we find that they do not significantly disrupt the chain-chain interactions, compared to unliganded simulation.

## Introduction

Up to 3% of the global population over 65 years old is affected by Parkinson's disease (PD), the second leading neurodegenerative disorder after Alzheimer's disease (AD). PD is a movement disorder of the dopaminergic neurons from the substantia nigra^1,2^. α-synuclein (α-Syn) is a well-known protein that is misfolded in PD, the main constituent of aggregates that form Lewy bodies (LBs). α-Syn controls dopaminergic neurotransmission and is involved in soluble N-ethylmaleimide-sensitive factor-attachment protein receptor (SNARE-complex) assembly^3–5^, although its function has yet to be fully discovered. α-Syn is very abundant in the brain, with ~ 1% of total rat brain proteins being α-Syn. It is also found in the heart, lungs, muscles, kidneys, and red blood cells^3,6^. The term "synuclein" refers to its localization in the synapses and nuclear envelope. Lewy bodies are major neuropathological characteristics of PD and dementia, and α-Syn is also the critical component that forms pale bodies and Lewy neurites (LNs)^7–9^. α-Syn aggregates appear either as plaques for extracellular deposition or inclusions for intracellular bodies, commonly observed in amyloid disorders such as PD ^10^. Decades of studies have examined the conformational changes, aggregates, and testing of α-Syn in search of ways to stop, lower the rate, or reverse PD-associated α-Syn aggregation^4,11,12^. Generally, any clinical condition initiated (thermodynamically) via inappropriate protein folding that leads to a high β-sheet content and fibrillar characteristics compared to its natural soluble form is called amyloidosis^4,13,14^. The inclusion of α-Syn, mainly seen in LB and LN, is called synucleinopathy; this is abundant with β-sheet aggregates^11^. α-Syn is an *SNCA* gene product; in humans, it is expressed as a 140 amino acid protein in neurons' presynaptic terminals^15^. With 60 amino acids at N-ter, nonamyloid-β component, which is primarily hydrophobic and composed of amino acids 61–95 that connect the N-ter to the acidic C-ter from 96 to 140^6,16^ (Supp Fig. S1). The lipid binding domain is located within the first 100 aa at the N-terminus and is composed of seven imperfect repeats of 11 aa. The main domain for its aggregation is the hydrophobic domain which is considered for investigation in this study^17^. Available treatments, such as cholinesterase, dopamine agonist, and monoamine oxidase, have not been fully effective; furthermore, their potential decreases during disease progression^18,19^. In contrast to soluble and highly disordered (yet functional) monomeric α-Syn in healthy individuals, oligomers, protofibrils, and LB aggregates are highly organized, ordered, and insoluble that align into compact β-sheet structures^20^. Studies to date have mainly tried to address the α-syn monomer and its process of aggregation, potential chemicals for preventing/reversing aggregation, and its conformational plasticity^21,22^. However, the exact role and interactions of each deformed and conformationally modified chain of α-syn remain uncertain with respect to each other and whole oligomers. Studying the dynamics of these chains and whole oligomers might yield useful information to design drugs or antibodies against aggregates^23–26^. The size of α-Syn protofibrils is approximately 7 nm, ~ 3 nm less than mature fibrils, which in vitro measurements have revealed to be approximately 10 nm^27^. Encountering aggregate generation is impossible without adequate knowledge of the atomistic and large-scale structural motions of the forming atoms and their interactions with other aggregate components^28^. Any small molecule, peptide, or antibody that reduces the aggregation rate or affects oligomer conformation to induce the generation of nonpathogenic species may be considered a possible strategy for tackling this problem^29^. Dozen small molecules, including phytochemicals, exhibit some neuroprotective effects, particularly in inhibiting the fibril formation of α-Syn through redirection of the α-Syn fibrils toward non-amyloidogenic structures^30^. Recent research has indicated that natural dyes such as Brazilin, curcumin, and crocin are highly effective in preventing the formation of α-Syn fibrils^31–33^. In several studies, the role of EGCG ((-)-epigallocatechin-3-gallate), the biologically active compound found in green tea, has been extensively investigated on both amyloid and α-Syn, and revealed that EGCG possesses the capacity to interfere with the β-sheet structure seen in the amyloid polypeptide and α-Syn or attenuates α-synuclein protofibril-membrane interactions^21,22,34–40^. In this study, we select the most ordered core of the protein in an oligomer arrangement with six chains. We observe all motions of these six chains without any constraints to evaluate their flexibility and the role of the chemical compounds that may reduce or even increase flexibility. We seek to find the most energetic and weak interacting regions, identify the effective residues in weak or strong regions, and determine the most potential target site for designing drugs or nanobodies to weaken their interactions. The helix conformation of α-Syn is mostly observed when it is in its functional form and interacting with vesicles and membranes^41^; otherwise, it undergoes conformational changes to lose the necessary interactions required for helix stabilization in favor of β-sheet strand, aggregate, fibril, and ultimately, LB formation^3^. The exact atomistic details of protein inclusion and α-syn aggregation are not yet fully understood. However, some studies show that LB formation begins with the monomeric form of α-Syn (i.e., seeds), which extend into twisted amyloid fibrils before developing fully into mature fibrils^3,42^. It is estimated that number of oligomer chains required to initiate the larger species, and trigger the seeding capacity to > 15^42^. We aim to study the dynamics of oligomers with fewer chains to uncover what occurs when fewer chains interact with each other or with a chemical compound.

## Materials and methods

### Docking simulation

Three chemical compounds were selected for study of their interactions and impact on the stability of α-Syn. C14, or (4-((R)-2-((R)-2,2,2-trifluoro-1-hydroxyethyl)pyrrolidin-1-yl)-2-trifluoromethyl) benzonitrile; gingerol, or (5S)-5-hydroxy-1-(4-hydroxy-3-methoxyphenyl)decan-3-one (Gin)); and C10, or 4-(trifluoromethyl) cinnamaldehyde.

Pujols et al.^43^ reported that small lipophilic molecules with trifluoromethyl groups on their structures are promising candidates for PD treatment. For this reason, we screened similar compounds and selected two synthetic compounds (C10 and C14) for molecular dynamics simulations, in addition to one naturally occurring compound. There is much experimental evidence of ginger's beneficial effects on PD development^44^. Thus, we consider gingerol (the main pharmaceutically active compound in ginger) in our simulations.

**Figure 1:**
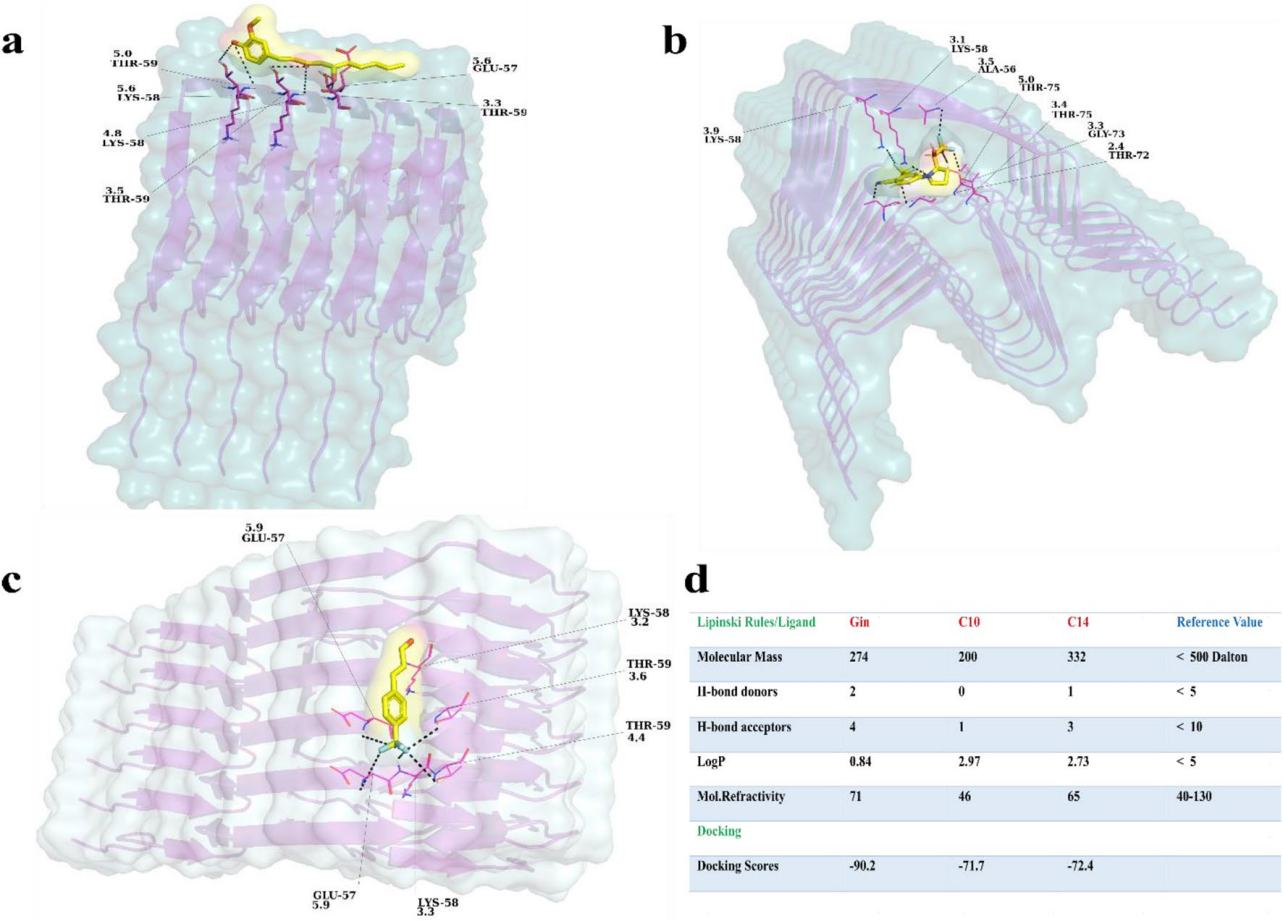
Docking simulation of Gin, C14, and C10 and assessment of their drug-likeness. (**a**) Docking of Gin and its most energetic pose with a score of  −90.2. (**b**, **c**) Docking of C14 and C10 with scores of  −72.4 and  −71.7, respectively. In all dockings, the ligand is yellow with a light surface and a stick representation; residues at a distance of < 6 Å are in magenta (stick shape). (**d**) Evaluation of "Lipinski's rule of five" for the three ligands and their docking scores^57,58^.

To model the interaction between ligands (4-R-2-((R)-2,2,2-trifluoro-1-hydroxyethpyrrolidinedin-1-yl)-2-trifluoromethyl) benzonitrile (C14) and (5S)-5-hydroxy-1-(4-hydroxy-3-methoxyphenyl) decan-3-one (Gin)), 4-(trifluoromethyl) cinnamaldehyde (C10), and the α-Syn fiber, we obtained the cryo-electron microscopy structure of the α-Syn fiber (PDB: 6A6B) from the Protein Data Bank, (http://www.pdb.org) as a target for the docking and MD simulations. The geometries of the ligands are shown in Fig. 1a–c. iGemdock v2. 1 docking tool was used to find the most energetic poses between compounds and the α-Syn^45^. This tool evaluates the hydrogen-bonding (H), van der Waals (V), and electrostatic (E) interactions. We estimated the empirical scoring function of iGemdock as follows: binding energy = vdW + Hbond + Elec (Fig. 1d). The calculated binding energy for C14 and α-Syn fiber was  −72.4 in a unit of the software's score, including the contributions of hydrogen and van der Waals bonding  −10.0 and  −62.40, respectively. Similar parameters were calculated for Gin and C10, the binding energy was  −90.24, and the contributions of hydrogen and van der Waals were −19.32 and  −70.92, respectively for Gin. For C10, the binding energy was  −71.72, the van der Waals contribution for this complex was  −65, and the hydrogen contribution was  −6.70.

### Molecular dynamics simulation

The Molecular Dynamics simulations (MDs) of the oligomer structure of α-syn in interactions with three chemical compounds and one unliganded simulation were performed for 300 ns (ns) using 2021.1 GROMACS^46,47^, and GROMOS96 54A7 force field^48,49^. The GROMOS96 G54A7FF United-Atom topologies (ITP file) for all three compounds were obtained from Automated Topology Builder (ATB) and Repository^50^. A dodecahedron boxes with periodic boundary conditions and 1.0 nm distances between the box edge and complexes was defined before SPC water models filled it out. Systems were neutralized and energy minimized by the steepest descent minimization algorithm. To avoid atomic clashes before starting the simulation, and after minimization, all conformations were compared with the initial coordinates. The time step for each simulation was set to 2 fs, and the "md" integrator was employed to integrate Newton's equations of motion. The electrostatic (long-range) and van der Waals (short-range) interactions were treated by Particle Mesh Ewald^51^ and Lennard Jones, respectively, while applying a 1.2 nm cutoff. The temperature and pressure were kept stable at 300 k and 1 bar by assigning a modified Berendsen thermostat^52,53^ (V-rescale) applying time constant of τ_t = 0.1 ps and Parrinello-Rahman pressure coupling with the compressibility of 4.5e−5^54^. All simulations used the LINCS algorithm as the constraint algorithm for bond length. Before the production simulation for 300 ns, the Position Restrain (PR, NVT ensemble with constant Number of particles, Volume, and Temperature) and NPT simulation (NPT ensemble which is constant for Number of particles, Pressure, and Temperature) were conducted for one ns and five ns, respectively, with the same 2 fs time step. The five ns of NPT simulation was performed after the NVT simulation. PyMOL (The PyMOL Molecular Graphics System, Version 2, Schrödinger, LLC), Grace (https://plasma-gate.weizmann.ac.il/Grace/) and VMD (https://www.ks.uiuc.edu/Research/vmd/), were used for visualization or generating graphs.

### Free energy evaluation

Each compound's free binding energy was calculated through the *g_mmpbsa* tool from 250 to 300 ns. The *g_mmpbsa* is applying molecular mechanics Poisson–Boltzmann surface area (MM-PBSA) to evaluate free energies^55,56^.

The binding free energy is measured as.

1 $$\Delta {\text{G}}_{{{\text{binding}}}} = {\text{G}}_{{\alpha {\text{ - Syn}}{-}{\text{Compounds}}}} {-} \, \left( {{\text{G}}_{{\alpha {\text{ - Syn}}}} + {\text{ G}}_{{{\text{Compounds}}}} } \right)$$

The total energy between α-Syn and ligands can be seen as “G_α-Syn–Compounds”_. The free energy, in general, for each entity was evaluated according to Eq. (2)^55^.

2 $${\mathrm{G}}_{L} =\langle {E}_{MM}\rangle -\mathrm{ TS }+\langle {G}_{Solvation}\rangle$$

The L indicates α-Syn-Compounds, and 〈E_MM〉 measures average vacuum potential energy, which is the potential energy for bonded and nonbonded interactions. The nonbonded interactions of electrostatic, and van der Waals were calculated using the *g_mmpbsa* tool as well. The bonded interaction measures the contribution of improper, angles, bonds, and dihedrals. The entropy (S) and temperature (T) are evaluated as the TS in Eq. (2) and refers to entropic contribution to free energy in a vacuum. The average free energy of solvation as it requires to move solute from a vacuum into the solvent counts as $\langle {G}_{Solvation}\rangle$. Notably, the *g_mmpbsa* solvent is not explicit, and the free energy of solvation includes polar and nonpolar solvents^55^.

## Result and discussion

To further address the lipophilicity and trifluoromethyl of our compounds, we have evaluated the ADME (Absorption, distribution, metabolism, and excretion) parameters, pharmacokinetic properties, druglike nature and medicinal chemistry friendliness using SwissADME (Supp Fig. S2)^59^. The general assessment has shown that Gin has strong physicochemical properties, and is able to pass the BBB (blood-brain barrier), and its GI absorption is relatively high according to the Boiled-Egg as reference. In terms of drug-likeness, it passes all filters, such as Ghose, Veber, Egan, Muegge, and its bioavailability score is 0.55. As it has been accepted, the standard way of lipophilicity is (log *P o/w*), which is the partition coefficient between n-octanol and water. The tool uses the consensus Log *P o/w* as the average of five other methods. The Lipophilicity for Gin is 3.13, which is in the optimal range (Supp Fig. S2)^60^. The lipophilicity of the C10 is 3.06; similar to Gin it is BBB permeant and with high GI absorption, and both molecules, as explained, did not violate the Lipinski's rule of five. For C14, almost all descriptors such as water solubility, Pharmacokinetics, drug-likeness, Physicochemical and especially Lipophilicity meet the accepted criteria. The Log *P o/w* is 3.57 (Supp Fig. S2)^61^.

**Figure 2:**
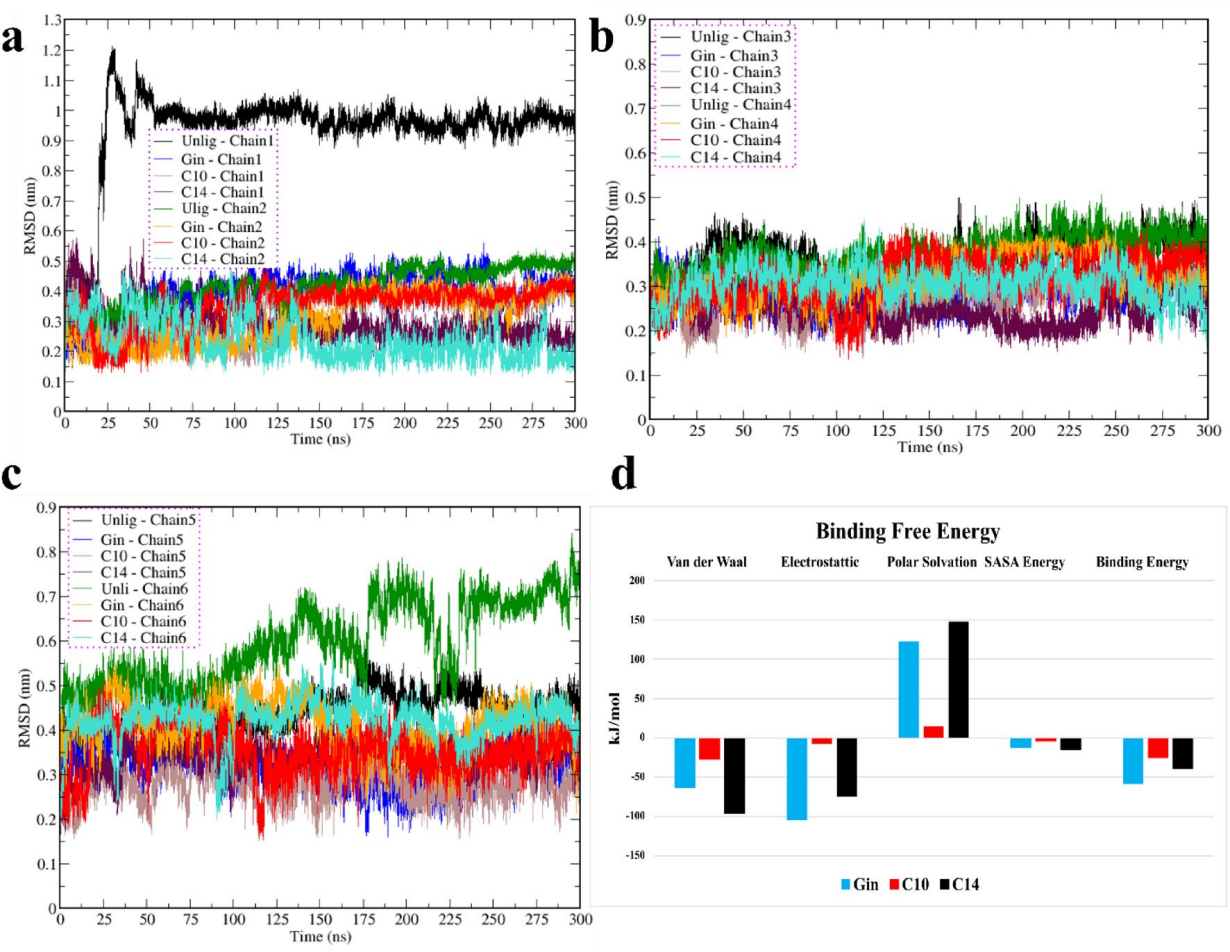
The root mean square deviations (RMSDs) of the α-Syn chains. (**a**) Chains 1 and 2; (**b**) chains 3 and 4; (**c**) chains 5 and 6. (**d**) Summary of binding energy calculated using the *g_mmpbsa* tool in GROMACS. In all figures (**a**–**c**), black, blue, brown, maroon, green, orange, red, and turquoise are used to display chains 1 through 2, 3 through 4 and 5 through 6.

To see the stability of each chain in different simulations the root mean square deviation (RMSD) was calculated. The reference structure for comparison was set to the widely accepted minimized equilibrated conformation. The backbone atoms of chains 1 and 6 (belonging to an unliganded oligomer) showed a higher value of RMS deviation for chain 1 (~ 10 Å) and a nonconverged value of ~ 5.5 to 8 Å from 155 ns for chain 6 (Fig. 2a,c). It was unclear whether this could be attributed to more dimensional freedom of the unliganded simulation or not. We did not intend to claim whether our compounds would have any effctive therapeutic impact in PD, but rather sought to study their interactions on the chain's stability. Compounds were selected if they or their root compounds previously showed some impact on α-Syn or other proteins; all those selected had strong drug-likeness parameters, as revealed through the evaluation of Lipinski's "rule of five" (Fig. 1d).

The RMSDs for chains 1 and 2 of the three simulations (Gin, C10, and C14) showed convergence and stable behavior from 2 Å to a maximum of 5 Å, which is considered as an acceptable range. The lowest RMSD for chain 2 was seen for C14, from 150 to 300 ns at the range of 2 Å to 3 Å. Chains 3 and 4 for the unliganded and all liganded simulations fluctuated in the range of 2 Å to 4.5 Å, and larger RMSDs were observed for both chains in the unliganded simulation. The unliganded chain 5 (with ~ 5 Å) and chain 6 of the C14 simulations were less stable than chains 5 and 6 in the C10 and Gin simulations. Chains 1 and 6 were the most unstable, thus, we further investigated the behaviors of these two chains in addition to four chains with liganded and unliganded simulations. (Fig. 2a–c).

Apart from the docking analysis and SwissADME, to ensure strong interaction between chemical compounds and α-Syn, we evaluated free energy through a detailed explanation in the material and methods (Section “Free energy evaluation” and Fig. 2d). The results confirmed that Gin has the most energetically favorable binding to the oligomer with  −58.3 kcal/mol compared to  −38.9 kcal/mol for C14 and  −25.6 kcal/mol for C10 (Fig. 2d). This result, combined with the docking scores and "Lipinski's rule of five" assessment, indicated strong binding between selected compounds and α-Syn oligomers.

**Figure 3:**
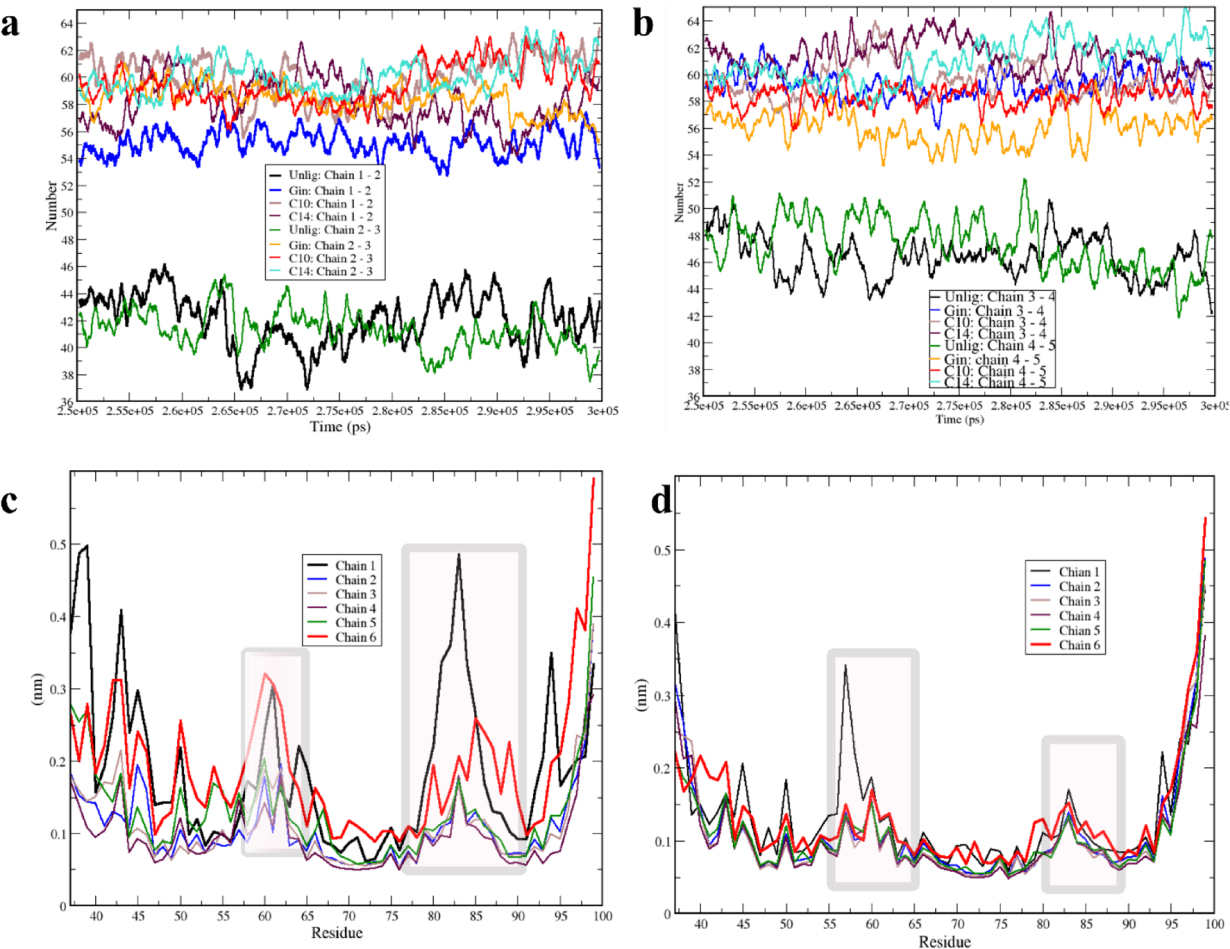
Hydrogen bonding between neighboring chains and their structural flexibility from 250 to 300 ns. (**a**) Number of H-bonds between chains 1 and 2 and chains 2 and 3 for the unliganded, Gin, C10, and C14 simulations are colored in black, blue, brown, and maroon between chains 1 and 2, respectively, and green, orange, red, and cyan between chains 2 and 3, respectively. (**b**) Numbers of H-bonds between chains 3 and 4 and chains 4 and 5 for the unliganded, Gin, C10, and C14 simulations are colored in black, blue, brown, and maroon for chains 3 and 4, respectively, and green, orange, red, and cyan for chains 4 and 5, respectively. (**c**, **d**) RMSFs for the 6 chains of unliganded and C14 simulations are colored in black, blue, brown, maroon, green, and red for chains 1 through 6, respectively.

We then evaluated hydrogen bonds at the cutoff of 3.5 Å between each neighboring chain to assess the H-bond numbers and insights into the roles of chemical compounds on chain instability. The number of H-bonds between chains 1 and 2 and between chains 2 and 3 in the unliganded system fluctuated between ~ 37 and ~ 44, while for all liganded simulations, they ranged from a minimum of ~ 52 to a maximum of ~ 62. We observed the same trend for H-bonds between chains 3 and 4 and between chains 4 and 5, as these ranged from ~ 43 to ~ 52 in the unliganded simulation and 54 to 64 in the liganded simulation. From these observations, we conclude that each chemical compound may contribute to more potent interactions among protofibril chains and that the three applied chemicals may stabilize them (Fig. 3a,b). The number of H-bonds between chains 5 and 6 in the unliganded simulation was 25 to 40 compared to the average of 50 to 69 in all other simulations and other chains (Supp Fig. S3).

By considering the RMSD and number of H-bonds, we can assume that larger structural motions occur in unliganded simulations. However, we are interested in knowing whether any trends exist within the different segments of the oligomer. Therefore, we examined the root mean square fluctuations (RMSFs) of the last 50 ns in the 24 chains for all simulations to uncover the nature of structural motion related to each chain, to determine the role of the chemical compounds, and to identify the segments with the most flexible chains. In the unliganded simulations, chains 1 and 6 showed RMS fluctuations of approximately 5 Å, without considering the residues at N or C-ter, which naturally are flexible especially if they are disordered (Fig. 3c). The regions with the most significant motions were in chains 1 and 6. Of these, chain 1 exhibited higher values primarily in three areas: aa 40 to 46, aa 56 to 63, and aa 79 to 84 (note that that segments are not necessarily loop). We view these regions where RMS fluctuation began to increase to a maximum fluctuation of ~ 4 and ~ 4.8 Å for chain 1, as well as ~ 3 Å and 2.5 Å for chain 6 for aa 40 to 46, and aa 79 to 84. Other chains (2 and 5) exhibited lower motion compared to chains 1 and 6 in the unliganded simulation, Gin and C10 (Fig. 3c,d and Supp Figs. S4–S5).

The chain fluctuation in C14 was lower than that in the unliganded simulation. Nevertheless, the trend of highly flexible regions resembled that of the unliganded simulation, with residues of aa 42 to 46, aa 56 to 63. Whether these flexible regions have a biological role or function as a critical protein segment has yet to be determined (Fig. 3d and Supp Figs. S4–S5).

**Figure 4:**
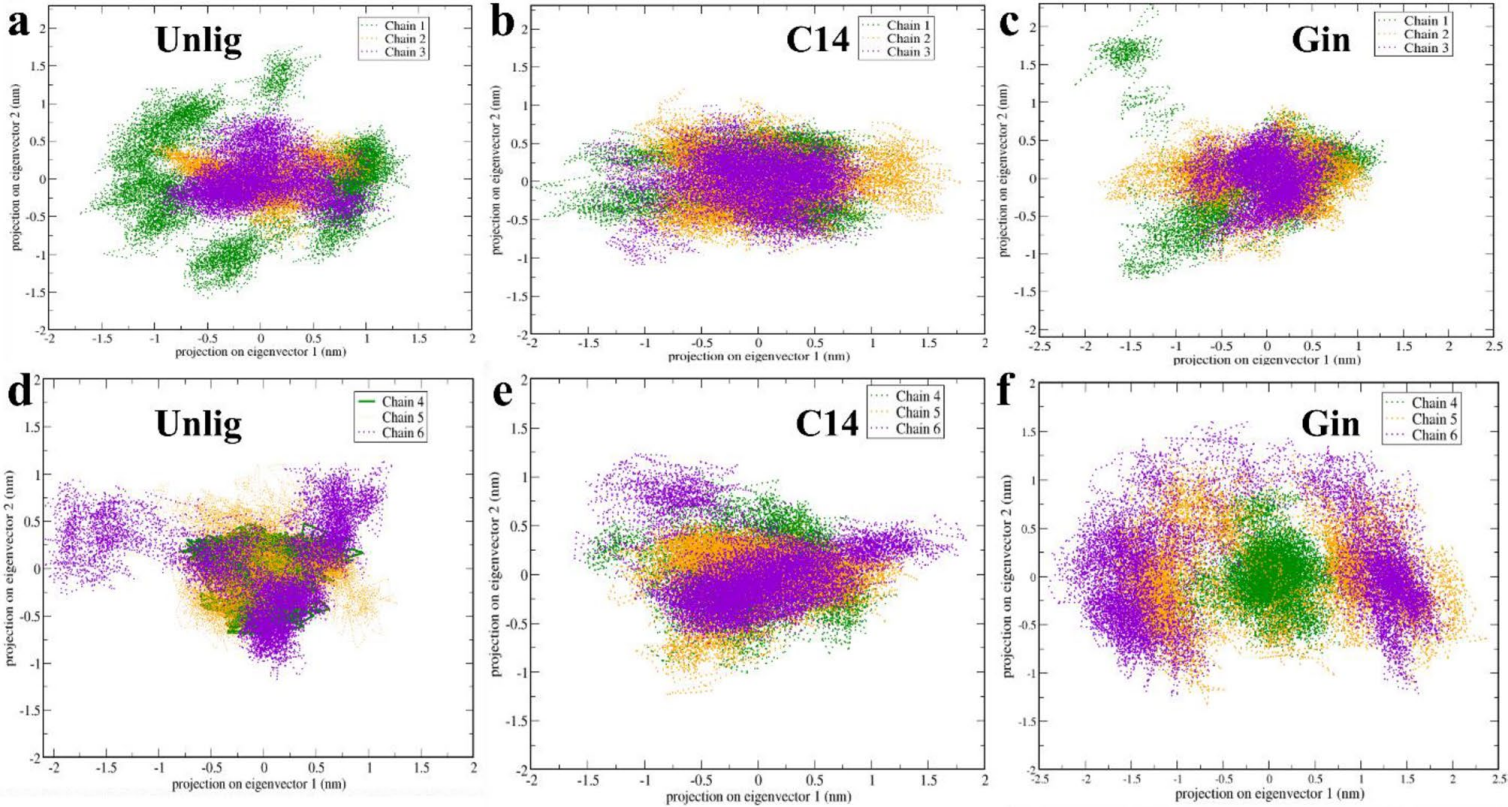
Principal component analysis. (**a**–**c**) Two-dimensional PC plot trajectory projection on the essential subspace for chains 1 through 3 for Unlig, Gin, and C14 are colored in green, orange, and violet for chains 1, 2 and 3, respectively. (**d**–**f**) Two-dimensional PC plot trajectory projection for chains 4 through 6 for Unlig, Gin, and C14 are colored in green, orange, and violet for chains 4, 5, and 6, respectively.

We then analyzed the protein's conformational dynamics and collective motion using essential dynamics (ED)^62^. We were able to calculate most of the converged conformations from 250 to 300 ns. The complexity of the generated data during MD simulation needed to be addressed with effective analysis to define the biological value of the conformational changes. The high-frequency localized motion of the proteins may be less crucial to the function than collective motions. We used principal component Analysis (PCA) to reduce the high-dimensional dataset onto the functional collective coordinates, as has been done successfully in several studies^62,63^. Covariance matrixes were generated from 250 to 300 ns for the Cα atoms in each chain. The diagonalization of the covariance matrix yielded the eigenvalues, and then the PC subspace spanned PC1 and PC2 for the unliganded, Gin, and C14 simulations for all six chains compared in each simulation (Fig. 4a–f).

A small number of PC vectors represented most of the movements of the chains. Here, we considered the first three PCs of the evaluated data. To observe the conformational dynamics of each trajectory along the eigenvectors, we projected the trajectories on the first two PCs. Chain 1 in the unliganded, Gin, and C14 simulations had more extended conformations that occupied a large phase space compared to chains 2 or 3 (Fig. 4a–c). However, their conformational behaviors were different; this was especially obvious when we compared the unliganded and two-liganded systems, which can further be extended to the role of the ligand. We observed more restricted motion in the essential subspace, which also correlated with lower RMS fluctuation, in chains 2 and 3. This was unsurprising because their motion was limited through the constrained sides of chains 1 and 4. In all three simulations, chains 1 and 6 mapped different areas of the principal plane, and chains 2 through 5 of the liganded systems occupied closer regions on the plane compared to those in the unliganded system (Fig. 4d–f).

**Figure 5:**
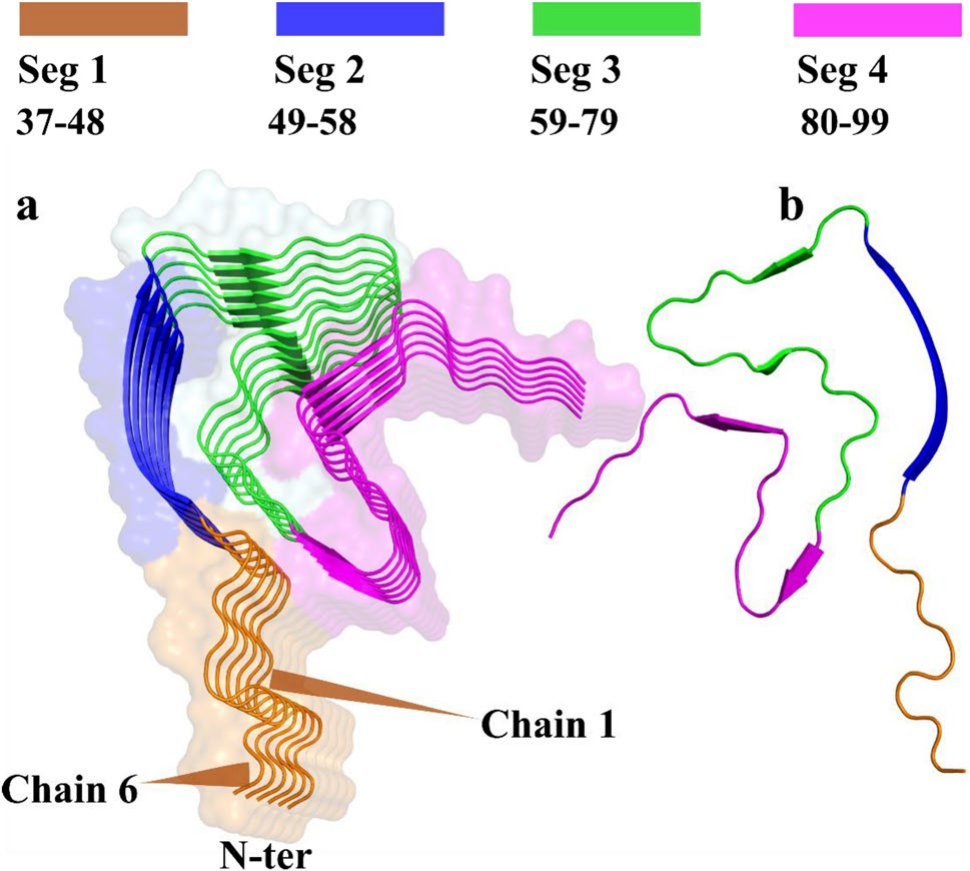
Four defined segments of α-Syn chains (PDB: 6A6B). In this study. We divided α-Syn into four fragments based on the degree of structural order or the point at which the chain initiated a shape turn. (**a**) The six oligomer chains of PDB: 6A6B crystal structure are divided into four segments from aa 37–aa 48 colored in orange, aa 49–aa 58 blue, aa 59–79 green and aa 80–aa 99 in magenta. (**b**) A single chain of the oligomer is displayed in the same color scheme as above. *aa* amino acids.

We then generated a heatmap to show the interaction energy from 250 to 300 ns in the Unlig, Gin, and C14 simulations to elucidate the energetic contributions of each chain residue with neighboring chains and to evaluate their roles in stabilizing the protein. In each chain (chains 1–6), all residues, one by one, are considered as one group and the neighboring chains as the second group. We then calculate the L–J and Coulomb interactions between each residue and its adjacent chain to obtain the interaction energy (IE), which is the sum of L–J and Coulomb. We divided α-Syn chains into four segments based on the extent to which they are ordered or where the shape turn began from the chain. Segment 1 encompassed aa 37 to aa 48, which were full loops; segment 2 includes aa 49 to aa 58, fully developed into a sheet conformation; and segments 3 and 4, which compose aa 59 to aa 79 and aa 80 to aa 99, respectively, with loop and small sheet conformations (Fig. 5).

The primary assessment showed that the IE among all liganded chains was higher than that of the unliganded chains (Table 1).

**Table 1:**
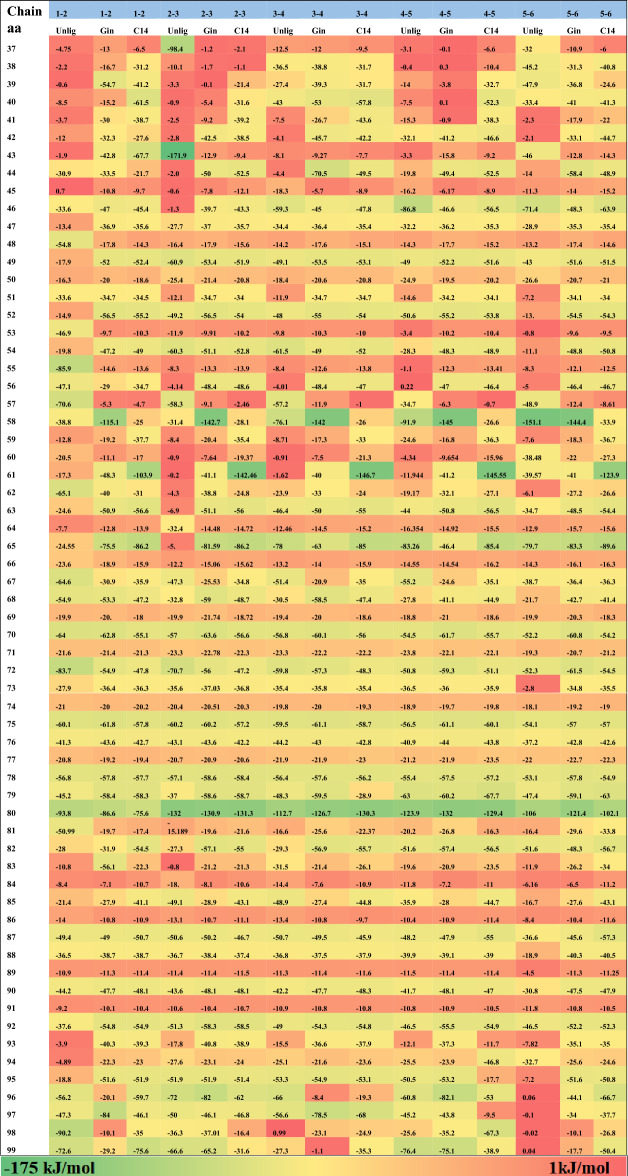
Heatmap of the IE between each residue (aa 37–aa 99) of chains 1, 2, 3, 4, 5, and 6, with respect to all adjacent chains (1, 2, 3, 4, 5, 6).

The IE is the sum of L–J and Coulomb energy between each residue and its adjacent chain. The color code ranges from green to yellow to red indicating highly negative to positive (as the strength of the IE is decreases). *aa* amino acids.

In chain 1, segment 1 had a lower IE value in the unliganded simulation for almost all residues apart from Val48, which strongly contributed to the binding to chain 2 and had an IE of  −54.8 kJ/mol. The IE between residues of chains 2–3, 3–4, 4–5, and 5–6 for segment 1 differed between the Gin and C14 simulations, but showed a similar trend in that most regions of the heatmap were reddish in color. The exceptions were four residues (37 through 40) of IE between the residues of chains 3–4 and 5–6, which interacted relatively strongly with their neighboring chains (Table 1). The sum of the IE (up to down) of all segments’ 1 residues between chains 1–2, 2–3, 3–4, 4–5, and 5–6 in the unliganded simulation were  −166.1,  −338.4,  −269.9,  −245.5, and  −347.9 kJ/mol, respectively. The weakest IE was between chains 1 and 2, and the most robust was between chains 5 and 6. Chain 1 only interacted in one direction with chain 2 and had more directional freedom than the other chains exhibiting different behaviors. The sums of the IE for segment 1 between chains 1–2, 2–3, 3–4, 4–5, and 5–6 in C14 were higher in all chains than in the unliganded simulation, apart from between chains 2–3 and higher than chains in Gin apart from between chains 3–4. This is noteworthy to mention that C14 is among the compounds that interact inside the protofibril.

Two residues that strongly contributed to stability in the interactions between the six chains, were Thr44 and Glu46, with sums of  −558.2 and  −736.1 kJ/mol, respectively, across all simulations, which were higher among all segment 1 residues in all three simulations (The cumulative sum across all simulations, from left to right). This might suggest that these two residues or the region that covers them can be a potential site of targeting, especially peptide-based or antibodies against α-Syn aggregates (Table 1).

In segment 2 (residues 49 through 58), some residues had lower (weak) IE in all simulations compared to others. For the six chains, His50, Ala53, Val55, and Glu57 (apart from Unlig) had very low IE at  −315.3,  −173.4,  −244.5, and  −332.5 kJ/mol (sum), respectively (The cumulative sum across all simulations, from left to right), if we exclude the contribution of Glu57 in the unliganded simulation at  −70.6 kJ/mol. Unsurprisingly, the sum of the IE of all the residues in segment 1 for the three simulations was  −4742.4, compared to  −5358.3 kJ/mol in segment 2 (sum the residues in each segment from top to bottom, and then sum them from left to right). These results indicate that the sheets in segment 2 are tied more strongly together, are among the significant points for fibril stability, and might represent the pivot points for the aggregate. In segment 3 (aa 59 through 79), small sheets and loops were the most significant constituents that made this segment the most challenging target because of its nature and drug accessibility. The sum of the IE for all chains in the three simulations for this segment was  −12,011.6 kJ/mol, which is higher than the sums for segments 1 and 2 at  −10,100.3 kJ/mol (segments 1 and 2 composed of 22 residues compared to 21 residues in segment 3). This shows that the combination of loop and sheet can strongly affect the strength of the interactions between chains. This segment and its residues may not be a reasonable target for the design of an effective therapy. Furthermore, in segment 3, the IE between chains (chains 1 and 2, 2 and 3, 3 and 4, 4 and 5, 5 and 6) was weaker in the unliganded simulation. The lowest IE values were observed between chains 2 and 3 at −596.22 kJ/mol, in Unlig compared to  −818.32 kJ/mol and  −917.47 kJ/mol in the Gin, and C14 simulations, respectively (Table 1).

Segment 4 comprised residues with a dominant loop-like shape. The sum of their IE in the three systems was −11,079.76 kJ/mol lower than that in segment 3. The weakest IE was observed at  −414.4 kJ/mol between chains 5 and 6 in the unliganded simulation. Residues of these segments in all three simulations and across all chains had very similar and weak IE; among them, the sums of Gly84, Gly86, Ala89, and Ala91 were  −150.3,  −168.1,  −164.1, and  −159.8 kJ/mol, respectively, for all chains in the three simulations (The cumulative sum across all simulations, from left to right).

## Discussion

Oligomers of small size and low molecular weight play an important role as a transient state in the generating of larger fibrils and aggregates. They are a transient and intermediate species, and reports exist of their toxicity^64^. Our research demonstrates that despite all chains having identical amino acid compositions, there were variations in the strengths of interactions between the chains within the four defined segments. This outcome is unsurprising, given the dynamic nature of proteins and the influence of various factors, including chemical agents, water, and ions. Nevertheless, the interpretation and comprehensive explanation of this dynamicity, to effectively benefit the design of a strategy for preventing the transition into full fibrils, require further exploration and discovery.

Applying and exposing the cells to drugs at very low concentrations do not necessarily induce destabilization of the α-Syn oligomers and aggregates or an effective impact^65^ even though there may have been some partial effects. It's worth noting that here, we only docked a single molecule, also, we must consider the limitations of computational studies and their compositions. Our evaluation of the structural motion of chains and the H-bonds between chains shows that the tested compounds generate fewer conformational motions and more H-bond interactions between oligomer chains. This may not be easily applicable to all chemicals as we only investigated three compounds.

We may have uncovered a trend among the more flexible residues (aa 44–46, aa 56–63, aa 82–84) in which they resembled flexible regions in the unliganded simulation. The evaluation of the conformational dynamics using the ED^62^ reveals that in the liganded simulations, the molecular behavior of chains differs from that in the unliganded simulation, possibly due to the influence of the chemical compounds. Nevertheless, these diverse conformations, which map differently at the plane, do not necessarily have weaker interactions among their chains, which might explain why there are still ineffective medications against Parkinson's disease or other neurodegenerative disorders. This study evaluated the contribution of 62 amino acids of all chains, Hence, incorporating all N-ter (N-terminal) and C-ter (C-terminal) residues might yield different outcomes and their exact roles must be investigated in different conditions.

In our study, we divided the core of the oligomer into four segments from the point where the loop changed to a sheet, or a sharp turn began in the conformation. Segments 1 to 4 were aa 37 through aa 48, aa 49 through aa 58, aa 59 through aa 79, and aa 80 through 99, respectively. Val48 exhibited the lowest IE (more negative or stronger interaction energy) in the unliganded simulation for all residues (apart from chain 1). This may have been the result of conformational freedom or compound impact. In the Gin and C14 simulations, chain 1 had similar dimensional freedom as in the unliganded simulation, but only in the unliganded simulation did it show its weakest interaction with chain 2 for Val48. The sum of the IE between neighboring chains was higher in the C14 simulation than in the unliganded one, with the exception of the IE between chains 2 and 3, and higher than in the Gin simulation apart between chains 3 and 4. Residues such as His50, Ala53, Val55, and Glu57 (apart from Unlig) for the six chains exhibited very low IE at  −315.3 kJ/mol,  −173.4 kJ/mol,  −244.5 kJ/mol, and  −332.5 kJ/mol (sum), respectively, if we exclude the contribution of Glu57 in the unliganded simulation at  −70.6 kJ/mol which showed the substantial contribution of the residue in the unliganded simulation for segment 2. In segment 3 (aa 59–79), the sum of the IE for all chains was higher than the sums in segments 1 and 2. Small sheets and loops were significant constituents that made it the most challenging target because of its nature and drug accessibility; we can conclude that drugs may be unable to penetrate this region easily. The sum of the IE for all chains in the three simulations for this segment was  −12,011.6 kJ/mol, which was higher than the sums of segments 1 and 2 at  −10,100.3 kJ/mol. This shows that the combination of loops and sheets can strongly affect the strength of the interactions between chains.

Kamelabad et al. conducted a study examining the impact of Curcumin (CUR) and Rosmarinic acid (RA) on disrupting the oligomer structure of PDB: 2N0A. In their research, they excluded all N-terminal (N-ter) and C-terminal (C-ter) residues to focus specifically on the NAC region^66^. Other studies have also utilized computational simulations, in conjunction with wet lab experiments or independently, to investigate the effects of various molecules on comparable or similar oligomers. In one study, Saffari et al. unveiled the influence of Crocin on α-Syn; the key residues engaged in interactions with the ligands, with a particular focus on the NAC region. Notably, residues 62–66, 70–75, 77–81, and 85–96 within the NAC region were found to be significant sites of interaction, which also have been explicitly considered in our study; however, our future studies will focus on evaluating the influence and significance of both terminal residues. It is worth mentioning that the results of binding free energy evaluation in our study exhibit a correlation with their research. For instance, the calculated free energy for C10 is  −25.6 kcal/mol, compared to  −23.93 kcal/mol for ligand-bound central protofibril in their work^33^. Guzzo et al. found two segments of the α-Syn exhibit a stronger tendency to aggregate during the initial phase of dimerization, including residues 36–55 and residues 66–95 (segments 1, 3 and 4 here). Importantly, our study also confirms the essential roles played by these residues in mediating chain-chain interactions^67^. This is noteworthy that inhibiting fibril formation might cause the oligomers enrichment, studies have indicated the possibility of toxicity with different species of α-Syn. Different levels of neurotoxicity relate to soluble and insoluble α-Syn species (oligomers, prefibrillar, and fully mature fibrils). All these species can be toxic, but the extent to which one disrupts cellular homeostasis and leads to neuronal death is under debate. Some studies indicate that mature fibrils are highly toxic due to their ability to interact and permeabilize cell membranes^42,68^. Other assessments asserted that oligomers are the more toxic species^69–71^.

We believe that conducting computational investigations before synthesis and wet lab testing significantly reduces the cost and time required for chemicals to reduce the density of the protein aggregates present in neurodegenerative disorders, especially PD. We conclude that candidates with extended aliphatic chains may be able to stabilize the non-bond interactions among chains and strengthen their interactions.

### Supplementary Information

Supplementary Figures.

## Data Availability

The datasets used and/or analyzed during the current study are available from the corresponding author on reasonable request.
