# Supplementary material for: Conformational dynamics of α-synuclein and study of its intramolecular forces in the presence of selected compounds
Source: Sci Rep. 2023 Nov 3;13:19020. doi: 10.1038/s41598-023-46181-1 (PMC10624887; doi:10.1038/s41598-023-46181-1)
Supplement: Supplementary file 1 — Supplementary Figures. [file 41598_2023_46181_MOESM1_ESM.docx]

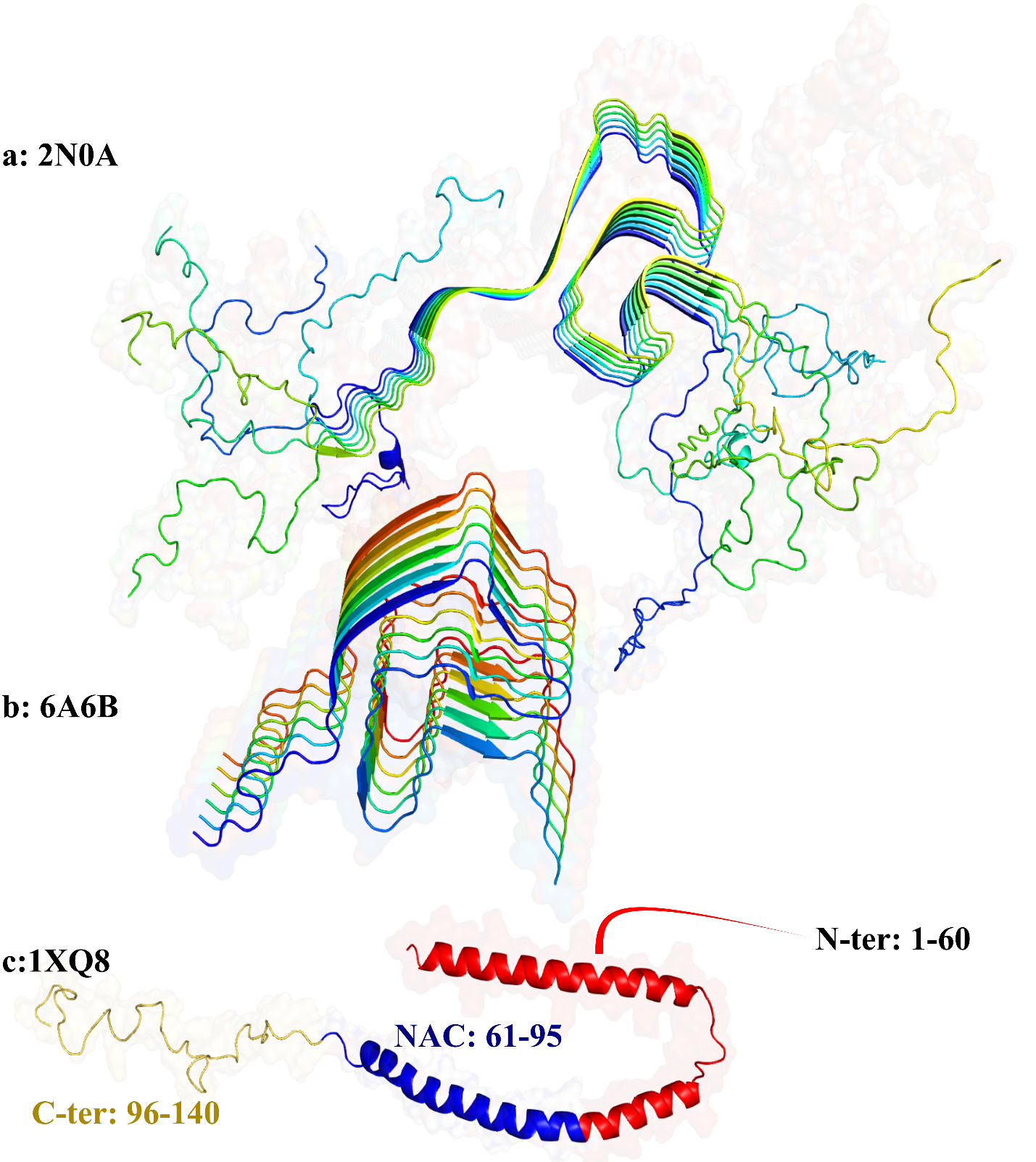


**Figure S1: The crystal strutures of α-Syn.** (**a-c**) are three different structures of α-Syn. We used six chains of PDB: 6A6B. The N-ter, NAC and C-ter of the single chain of the α-Syn is shown.

**
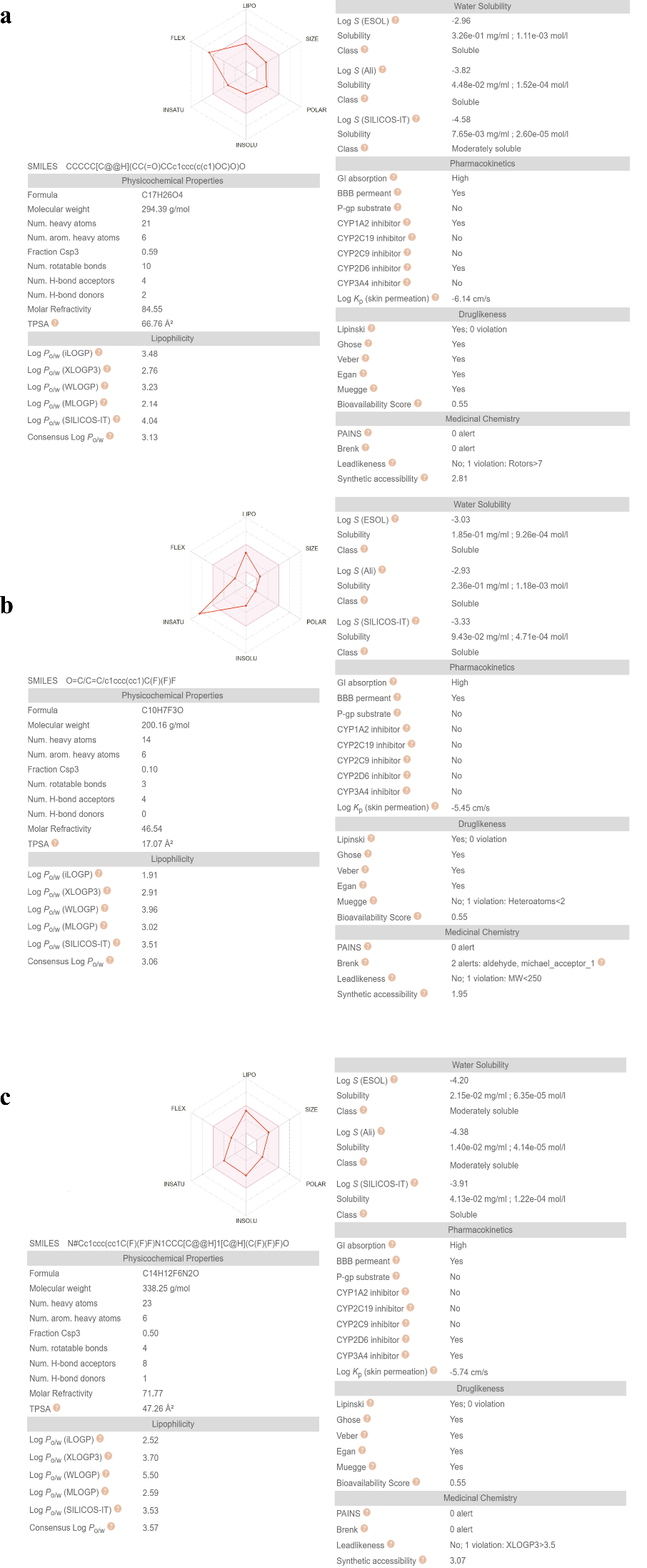
**

**Figure S2: (a-c) Evaluation of pharmacokinetics, drug-likeness and medicinal chemistry of Gin, C10 and C14 respectively.**


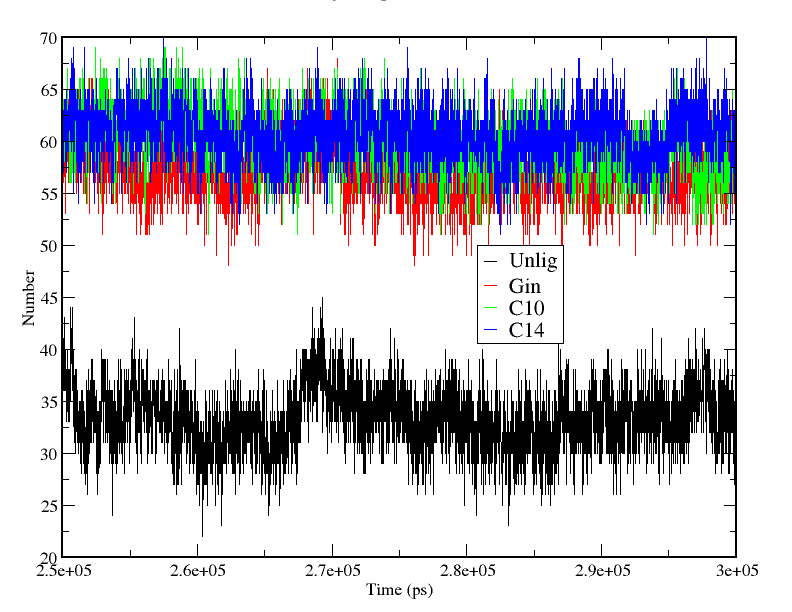


**Figure S3:**  Number of H-bonds between chains 5 and 6 for the unliganded, Gin, C10, and C14 simulations are colored in black, red, green, and blue, respectively.


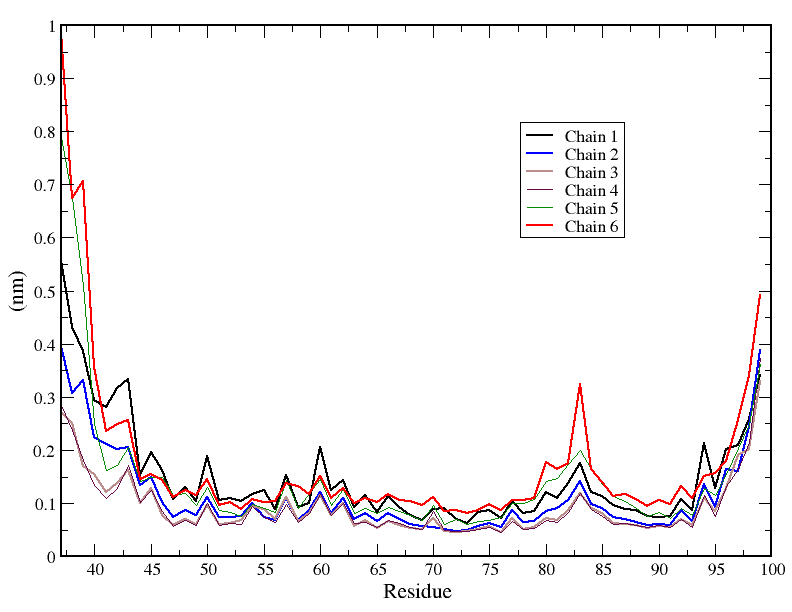


**Figure S4**: RMSF for the 6 chains of Gin simulation are colored in black, blue, brown, maroon, green, and red through chains 1 through 6, respectively.


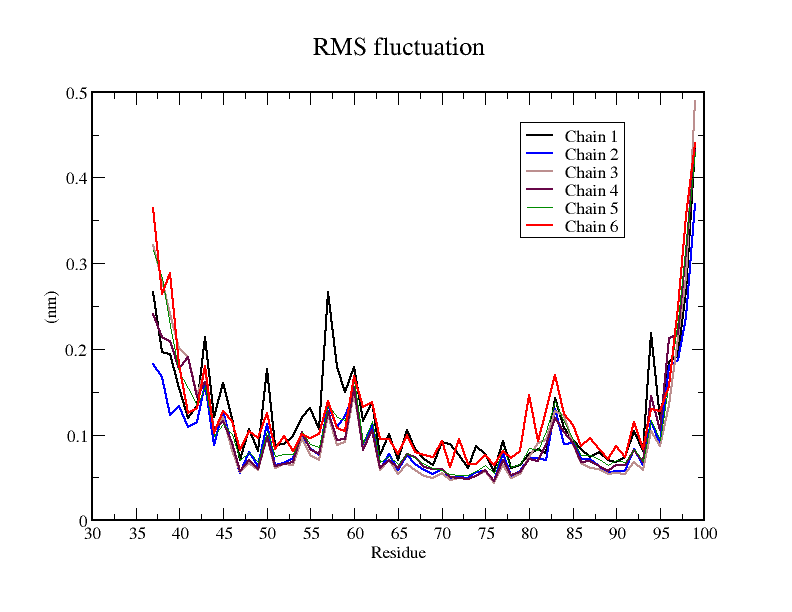


**Figure S5**: RMSF for the 6 chains of C10 simulation are colored in black, blue, brown, maroon, green, and red through chains 1 through 6, respectively.
